# Bis(2-amino-1,3-benzothia­zole-κ*N* ^3^)dichloridozinc(II) ethanol hemisolvate

**DOI:** 10.1107/S1600536812001560

**Published:** 2012-01-21

**Authors:** Young-Inn Kim, Sung Kwon Kang

**Affiliations:** a Department of Chemistry Education, Interdisciplinary Program of Advanced Information and Display Materials, and Center for Plastic Information System, Pusan National University, Busan 609-735, Republic of Korea; b Department of Chemistry, Chungnam National University, Daejeon 305-764, Republic of Korea

## Abstract

In the title compound, [ZnCl_2_(C_7_H_6_N_2_S)_2_]·0.5CH_3_CH_2_OH, the Zn^II^ atom is coordinated by two N atoms of two 2-amino­benzothia­zole ligands and two Cl atoms within a distorted tetra­hedral geometry. The dihedral angle between the N/Zn/N and Cl/Zn/Cl planes is 86.22 (7)°. The benzothia­zole mol­ecules are almost perpendicular to each other, forming a dihedral angle of 80.20 (8)°. The mol­ecular structure is stabilized by intra­molecular N—H⋯Cl hydrogen bonds. In the crystal, inter­molecular N—H⋯Cl hydrogen bonds link the mol­ecules into a three-dimensional network. The *SQUEEZE* procedure in *PLATON* [Spek (2009 ▶). *Acta Cryst.* D**65**, 148–155] was used to model a disordered ethanol solvent mol­ecule; the calculated unit-cell data allow for the presence of half of this mol­ecule in the asymmetric unit.

## Related literature

For the synthesis and structures of related Zn^II^ and Hg^II^ metal complexes, see: Kim *et al.* (2007 ▶, 2010 ▶, 2011 ▶); Seo *et al.* (2009 ▶); Kim & Kang (2010 ▶). For the biological and photochemical properties of benzothia­zole compounds, see: Khan *et al.* (2011 ▶); Pavlovic *et al.* (2007 ▶); Raposo *et al.* (2011 ▶); Saeed *et al.* (2010 ▶); Zajac *et al.* (2008 ▶).
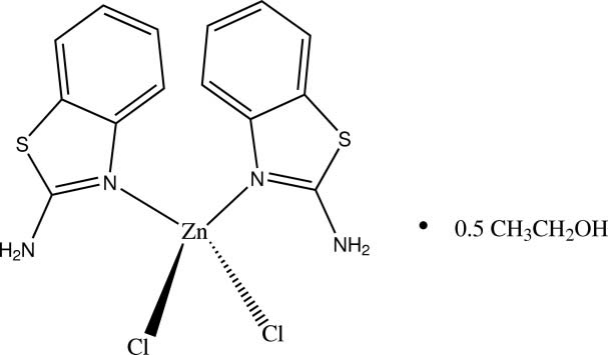


## Experimental

### 

#### Crystal data

[ZnCl_2_(C_7_H_6_N_2_S)_2_]·0.5C_2_H_6_O
*M* *_r_* = 459.72
Orthorhombic, 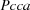

*a* = 21.0129 (10) Å
*b* = 11.6013 (5) Å
*c* = 16.6025 (8) Å
*V* = 4047.3 (3) Å^3^
*Z* = 8
Mo *K*α radiation
μ = 1.69 mm^−1^
*T* = 296 K
0.13 × 0.10 × 0.07 mm

#### Data collection

Bruker SMART CCD area-detector diffractometer
Absorption correction: multi-scan (*SADABS*; Bruker, 2002 ▶) *T*_min_ = 0.813, *T*_max_ = 0.881
16706 measured reflections
4956 independent reflections
2612 reflections with *I* > 2σ(*I*)
*R*_int_ = 0.058

#### Refinement

*R*[*F* ^2^ > 2σ(*F* ^2^)] = 0.046
*wR*(*F* ^2^) = 0.136
*S* = 0.88
4956 reflections
208 parameters
H-atom parameters constrained
Δρ_max_ = 0.39 e Å^−3^
Δρ_min_ = −0.34 e Å^−3^

### 

Data collection: *SMART* (Bruker, 2002 ▶); cell refinement: *SAINT* (Bruker, 2002 ▶); data reduction: *SAINT*; program(s) used to solve structure: *SHELXS97* (Sheldrick, 2008 ▶); program(s) used to refine structure: *SHELXL97* (Sheldrick, 2008 ▶); molecular graphics: *ORTEP-3* (Farrugia, 1997 ▶); software used to prepare material for publication: *WinGX* (Farrugia, 1999 ▶).

## Supplementary Material

Crystal structure: contains datablock(s) global, I. DOI: 10.1107/S1600536812001560/tk5044sup1.cif

Structure factors: contains datablock(s) I. DOI: 10.1107/S1600536812001560/tk5044Isup2.hkl

Additional supplementary materials:  crystallographic information; 3D view; checkCIF report

## Figures and Tables

**Table:** 

Zn1—N4	2.026 (3)
Zn1—N14	2.028 (3)
Zn1—Cl2	2.2489 (11)
Zn1—Cl3	2.2726 (11)

**Table:** 

N4—Zn1—N14	112.24 (12)
Cl2—Zn1—Cl3	112.11 (5)

**Table 2:** Hydrogen-bond geometry (Å, °)

*D*—H⋯*A*	*D*—H	H⋯*A*	*D*⋯*A*	*D*—H⋯*A*
N13—H13*A*⋯Cl2	0.86	2.46	3.273 (3)	157
N13—H13*B*⋯Cl3^i^	0.86	2.49	3.314 (3)	161
N23—H23*A*⋯Cl3	0.86	2.54	3.333 (3)	154
N23—H23*B*⋯Cl2^ii^	0.86	2.57	3.366 (3)	154

Symmetry codes: (i) 
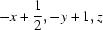
; (ii) 
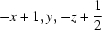
.

**Fig. 1.**
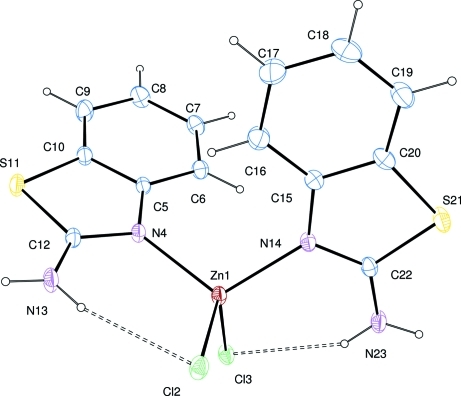
Molecular structure of (I), showing the atom-numbering scheme and 30% probability ellipsoids. Intramolecular N—H···Cl hydrogen bonds are indicated by dashed lines. The ethanol molecule is not shown.

**Fig. 2.**
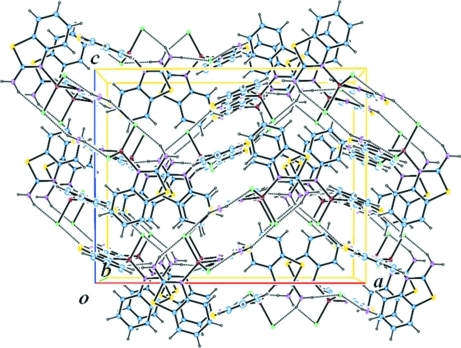
Part of the crystal structure of (I), showing molecules linked by intermolecular N—H···Cl hydrogen bonds (dashed lines). The ethanol molecule is not shown.

## supplementary crystallographic information

### Comment

Recently, we studied Zn(II) and Hg(II) complexes with nitrogen-containing ligands (Kim *et al.*, 2007; Seo *et al.*, 2009; Kim *et al.*, 2010; Kim & Kang, 2010; Kim *et al.*, 2011) with reference to their luminescent properties, as these can be used as fluorescent brighteners. As a part of our continuous interest in the coordination properties of the nitrogen-containing ligands, herein we report the synthesis of a Zn(II) chloride complex with 2-aminobenzothiazole, (I). Compounds with benzothiazole moiety are also of significant interest due to their biological properties such as anti-tumor and anti-viral (Saeed *et al.*, 2010; Khan *et al.*, 2011) as well as photochemical properties (Pavlovic *et al.*, 2007; Zajac *et al.*, 2008; Raposo *et al.*, 2011).

In (I), Fig. 1, the 2-aminobenzothiazole molecules are almost planar, with r.m.s. deviations of 0.022 and 0.009 Å from the corresponding least-squares plane defined by the ten constituent atoms, respectively. The Zn^II^ atom is coordinated by two N atoms of benzothiazole ligands and two Cl atoms in a distorted tetrahedral geometry with the dihedral angle of 86.22 (7)° between the N4/Zn1/N14 and Cl2/Zn1/Cl3 planes. The bond distances of N4—C12 [1.338 (4) Å] and N14—C22 [1.317 (4) Å] in the benzothiazole ligands are shorter than those of N4—C5 [1.404 (5) Å] and N14—C15 [1.395 (5) Å], respectively, which is consistent with double bond character in the former (Table 1). The benzothiazole molecules are almost perpendicular to each other with a dihedral angle of 80.20 (8)°. The molecular structure is stabilized by intramolecular N13—H13A···Cl2 and N23—H23A···Cl3 hydrogen bonds (Fig. 1 and Table 2). In the crystal, intermolecular N—H···Cl hydrogen bonds link the molecules into a three-dimensional network (Fig. 2).

### Experimental

All reagents and solvents were purchased from Aldrich and used without further purification. A mixture of ZnCl_2_ (0.66 g, 5.0 mmol) and 2-aminobenzothiazole (1.50 g, 10.0 mmol) in ethanol (20 ml) was stirred at room temperature under nitrogen atmosphere. The resulting colourless solution was allowed to stand at room temperature for a week to yield colourless crystals (yield 60.0%) suitable for X-ray diffraction.

### Refinement

All H atoms were positioned geometrically and refined using a riding model, with C—H = 0.93 Å and N—H = 0.86 Å, and with *U*_iso_(H) = 1.2*U*_eq_(C or N). There is a disordered ethanol solvent molecule which was difficult to model. Therefore, the *SQUEEZE* command of *PLATON* (Spek, 2009) was used to model the electron density in the void regions. There are two cavities of 378 Å^3^ per unit cell. Each cavity contains approximately 58 electrons which were assigned to two solvent ethanol molecules. With *Z* = 8, each Zn complex has 0.5 solvent ethanol equivalent. The reported molecular formula and derived unit cell characteristics take into account the presence of the solvent molecules.

### Crystal data

**Table:**

[ZnCl_2_(C_7_H_6_N_2_S)_2_]·0.5C_2_H_6_O	*F*(000) = 1760
*M**_r_* = 459.72	*D*_x_ = 1.509 Mg m^−^^3^
Orthorhombic, *P**c**c**a*	Mo *K*α radiation, λ = 0.71073 Å
Hall symbol: -P 2a 2ac	Cell parameters from 1986 reflections
*a* = 21.0129 (10) Å	θ = 3.0–24.3°
*b* = 11.6013 (5) Å	µ = 1.69 mm^−^^1^
*c* = 16.6025 (8) Å	*T* = 296 K
*V* = 4047.3 (3) Å^3^	Block, colourless
*Z* = 8	0.13 × 0.1 × 0.07 mm

### Data collection

**Table:**

Bruker SMART CCD area-detector diffractometer	2612 reflections with *I* > 2σ(*I*)
graphite	*R*_int_ = 0.058
φ and ω scans	θ_max_ = 28.3°, θ_min_ = 2.4°
Absorption correction: multi-scan (*SADABS*; Bruker, 2002)	*h* = −27→8
*T*_min_ = 0.813, *T*_max_ = 0.881	*k* = −15→12
16706 measured reflections	*l* = −19→21
4956 independent reflections	

### Refinement

**Table:**

Refinement on *F*^2^	Primary atom site location: structure-invariant direct methods
Least-squares matrix: full	Secondary atom site location: difference Fourier map
*R*[*F*^2^ > 2σ(*F*^2^)] = 0.046	Hydrogen site location: inferred from neighbouring sites
*wR*(*F*^2^) = 0.136	H-atom parameters constrained
*S* = 0.88	*w* = 1/[σ^2^(*F*_o_^2^) + (0.0724*P*)^2^] where *P* = (*F*_o_^2^ + 2*F*_c_^2^)/3
4956 reflections	(Δ/σ)_max_ = 0.001
208 parameters	Δρ_max_ = 0.39 e Å^−^^3^
0 restraints	Δρ_min_ = −0.34 e Å^−^^3^

### Special details

**Table:**

Geometry. All e.s.d.'s (except the e.s.d. in the dihedral angle between two l.s. planes) are estimated using the full covariance matrix. The cell e.s.d.'s are taken into account individually in the estimation of e.s.d.'s in distances, angles and torsion angles; correlations between e.s.d.'s in cell parameters are only used when they are defined by crystal symmetry. An approximate (isotropic) treatment of cell e.s.d.'s is used for estimating e.s.d.'s involving l.s. planes.
Refinement. Refinement of *F*^2^ against ALL reflections. The weighted *R*-factor *wR* and goodness of fit *S* are based on *F*^2^, conventional *R*-factors *R* are based on *F*, with *F* set to zero for negative *F*^2^. The threshold expression of *F*^2^ > σ(*F*^2^) is used only for calculating *R*-factors(gt) *etc*. and is not relevant to the choice of reflections for refinement. *R*-factors based on *F*^2^ are statistically about twice as large as those based on *F*, and *R*- factors based on ALL data will be even larger.

### Fractional atomic coordinates and isotropic or equivalent isotropic displacement parameters (Å^2^)

**Table:**

	*x*	*y*	*z*	*U*_iso_*/*U*_eq_	
Zn1	0.387172 (17)	0.61071 (4)	0.10299 (3)	0.04953 (16)	
Cl2	0.33663 (4)	0.61006 (11)	0.22239 (6)	0.0701 (3)	
Cl3	0.42382 (4)	0.43298 (9)	0.06892 (7)	0.0598 (3)	
N4	0.32592 (12)	0.6503 (3)	0.01289 (18)	0.0477 (7)	
C5	0.34802 (15)	0.6653 (3)	−0.0662 (2)	0.0462 (8)	
C6	0.41141 (18)	0.6731 (4)	−0.0882 (2)	0.0569 (10)	
H6	0.4435	0.6673	−0.0499	0.068*	
C7	0.42565 (19)	0.6899 (4)	−0.1684 (3)	0.0624 (11)	
H7	0.4681	0.6945	−0.184	0.075*	
C8	0.3791 (2)	0.6999 (4)	−0.2255 (3)	0.0766 (13)	
H8	0.3899	0.7119	−0.2791	0.092*	
C9	0.3160 (2)	0.6922 (4)	−0.2033 (3)	0.0758 (13)	
H9	0.2841	0.6989	−0.2418	0.091*	
C10	0.30062 (17)	0.6744 (4)	−0.1237 (3)	0.0565 (10)	
S11	0.22578 (4)	0.66284 (11)	−0.07852 (7)	0.0681 (3)	
C12	0.26227 (15)	0.6488 (3)	0.0152 (2)	0.0519 (9)	
N13	0.22837 (13)	0.6396 (3)	0.0822 (2)	0.0682 (10)	
H13A	0.2473	0.6342	0.128	0.082*	
H13B	0.1875	0.6391	0.0799	0.082*	
N14	0.45956 (13)	0.7257 (3)	0.11147 (19)	0.0510 (8)	
C15	0.45232 (18)	0.8447 (4)	0.1039 (2)	0.0563 (10)	
C16	0.3996 (2)	0.9016 (4)	0.0752 (4)	0.0839 (15)	
H16	0.3638	0.8601	0.0595	0.101*	
C17	0.3998 (3)	1.0218 (5)	0.0695 (4)	0.1028 (18)	
H17	0.3648	1.0607	0.0486	0.123*	
C18	0.4536 (4)	1.0833 (5)	0.0957 (4)	0.1039 (19)	
H18	0.4536	1.1635	0.0936	0.125*	
C19	0.5060 (3)	1.0260 (5)	0.1243 (4)	0.0920 (16)	
H19	0.542	1.0665	0.1408	0.11*	
C20	0.5045 (2)	0.9079 (4)	0.1283 (3)	0.0689 (12)	
S21	0.56461 (5)	0.81677 (11)	0.16229 (8)	0.0794 (4)	
C22	0.51607 (16)	0.6994 (4)	0.1405 (2)	0.0526 (9)	
N23	0.53666 (14)	0.5938 (3)	0.1541 (2)	0.0673 (10)	
H23A	0.5125	0.5358	0.1439	0.081*	
H23B	0.5742	0.583	0.1732	0.081*	

### Atomic displacement parameters (Å^2^)

**Table:**

	*U*^11^	*U*^22^	*U*^33^	*U*^12^	*U*^13^	*U*^23^
Zn1	0.0339 (2)	0.0592 (3)	0.0555 (3)	−0.00259 (18)	−0.00195 (16)	−0.0009 (2)
Cl2	0.0450 (5)	0.1092 (9)	0.0562 (6)	0.0047 (5)	0.0029 (4)	0.0017 (6)
Cl3	0.0395 (4)	0.0593 (6)	0.0807 (7)	0.0003 (4)	0.0012 (4)	−0.0048 (5)
N4	0.0336 (13)	0.0584 (19)	0.051 (2)	−0.0019 (13)	−0.0030 (12)	−0.0022 (15)
C5	0.0385 (17)	0.044 (2)	0.056 (2)	−0.0035 (15)	−0.0026 (15)	−0.0041 (18)
C6	0.047 (2)	0.063 (3)	0.062 (3)	0.0010 (18)	0.0038 (17)	−0.002 (2)
C7	0.056 (2)	0.069 (3)	0.062 (3)	−0.0080 (19)	0.013 (2)	−0.010 (2)
C8	0.078 (3)	0.098 (4)	0.053 (3)	−0.019 (3)	0.008 (2)	−0.006 (3)
C9	0.070 (3)	0.103 (4)	0.055 (3)	−0.015 (3)	−0.015 (2)	−0.006 (3)
C10	0.043 (2)	0.066 (3)	0.060 (3)	−0.0058 (18)	−0.0032 (16)	0.000 (2)
S11	0.0404 (5)	0.0954 (9)	0.0686 (7)	−0.0065 (5)	−0.0123 (4)	0.0040 (6)
C12	0.0363 (17)	0.057 (2)	0.063 (3)	0.0000 (15)	−0.0028 (16)	−0.003 (2)
N13	0.0321 (15)	0.108 (3)	0.064 (2)	−0.0072 (16)	0.0005 (14)	0.001 (2)
N14	0.0403 (15)	0.053 (2)	0.059 (2)	−0.0049 (14)	−0.0028 (13)	−0.0030 (15)
C15	0.054 (2)	0.056 (3)	0.059 (3)	−0.0036 (19)	0.0036 (18)	−0.002 (2)
C16	0.080 (3)	0.062 (3)	0.109 (4)	−0.001 (2)	−0.017 (3)	−0.006 (3)
C17	0.102 (4)	0.070 (4)	0.136 (5)	0.018 (3)	−0.015 (4)	−0.004 (3)
C18	0.129 (5)	0.055 (3)	0.128 (5)	−0.008 (3)	0.014 (4)	−0.007 (3)
C19	0.083 (4)	0.074 (4)	0.119 (5)	−0.018 (3)	0.000 (3)	−0.010 (3)
C20	0.076 (3)	0.054 (3)	0.077 (3)	−0.015 (2)	0.008 (2)	−0.009 (2)
S21	0.0532 (6)	0.0765 (8)	0.1084 (10)	−0.0193 (5)	−0.0102 (6)	−0.0122 (7)
C22	0.0419 (19)	0.063 (3)	0.053 (2)	−0.0077 (18)	0.0060 (16)	−0.005 (2)
N23	0.0423 (16)	0.068 (3)	0.092 (3)	0.0004 (16)	−0.0166 (17)	−0.005 (2)

### Geometric parameters (Å, °)

**Table:**

Zn1—N4	2.026 (3)	N13—H13A	0.86
Zn1—N14	2.028 (3)	N13—H13B	0.86
Zn1—Cl2	2.2489 (11)	N14—C22	1.317 (4)
Zn1—Cl3	2.2726 (11)	N14—C15	1.395 (5)
N4—C12	1.338 (4)	C15—C16	1.374 (6)
N4—C5	1.404 (5)	C15—C20	1.380 (6)
C5—C10	1.384 (5)	C16—C17	1.399 (7)
C5—C6	1.384 (5)	C16—H16	0.93
C6—C7	1.378 (5)	C17—C18	1.405 (8)
C6—H6	0.93	C17—H17	0.93
C7—C8	1.368 (6)	C18—C19	1.373 (8)
C7—H7	0.93	C18—H18	0.93
C8—C9	1.379 (6)	C19—C20	1.372 (6)
C8—H8	0.93	C19—H19	0.93
C9—C10	1.375 (6)	C20—S21	1.741 (5)
C9—H9	0.93	S21—C22	1.740 (4)
C10—S11	1.748 (4)	C22—N23	1.318 (5)
S11—C12	1.742 (4)	N23—H23A	0.86
C12—N13	1.326 (5)	N23—H23B	0.86
			
N4—Zn1—N14	112.24 (12)	C12—N13—H13A	120
N4—Zn1—Cl2	110.59 (8)	C12—N13—H13B	120
N14—Zn1—Cl2	107.17 (9)	H13A—N13—H13B	120
N4—Zn1—Cl3	103.73 (9)	C22—N14—C15	111.1 (3)
N14—Zn1—Cl3	111.09 (9)	C22—N14—Zn1	123.3 (3)
Cl2—Zn1—Cl3	112.11 (5)	C15—N14—Zn1	124.3 (2)
C12—N4—C5	111.0 (3)	C16—C15—C20	119.2 (4)
C12—N4—Zn1	127.7 (3)	C16—C15—N14	126.5 (4)
C5—N4—Zn1	120.6 (2)	C20—C15—N14	114.4 (4)
C10—C5—C6	120.4 (4)	C15—C16—C17	120.0 (5)
C10—C5—N4	114.7 (3)	C15—C16—H16	120
C6—C5—N4	125.0 (3)	C17—C16—H16	120
C7—C6—C5	118.2 (4)	C16—C17—C18	119.2 (5)
C7—C6—H6	120.9	C16—C17—H17	120.4
C5—C6—H6	120.9	C18—C17—H17	120.4
C8—C7—C6	121.8 (4)	C19—C18—C17	120.5 (5)
C8—C7—H7	119.1	C19—C18—H18	119.8
C6—C7—H7	119.1	C17—C18—H18	119.8
C7—C8—C9	119.8 (4)	C20—C19—C18	118.8 (5)
C7—C8—H8	120.1	C20—C19—H19	120.6
C9—C8—H8	120.1	C18—C19—H19	120.6
C10—C9—C8	119.5 (4)	C19—C20—C15	122.3 (5)
C10—C9—H9	120.2	C19—C20—S21	127.2 (4)
C8—C9—H9	120.2	C15—C20—S21	110.4 (3)
C9—C10—C5	120.3 (3)	C22—S21—C20	89.0 (2)
C9—C10—S11	129.5 (3)	N14—C22—N23	125.0 (3)
C5—C10—S11	110.2 (3)	N14—C22—S21	115.0 (3)
C12—S11—C10	89.70 (17)	N23—C22—S21	119.9 (3)
N13—C12—N4	124.2 (3)	C22—N23—H23A	120
N13—C12—S11	121.4 (3)	C22—N23—H23B	120
N4—C12—S11	114.4 (3)	H23A—N23—H23B	120

### Hydrogen-bond geometry (Å, °)

**Table:**

*D*—H···*A*	*D*—H	H···*A*	*D*···*A*	*D*—H···*A*
N13—H13A···Cl2	0.86	2.46	3.273 (3)	157
N13—H13B···Cl3^i^	0.86	2.49	3.314 (3)	161
N23—H23A···Cl3	0.86	2.54	3.333 (3)	154
N23—H23B···Cl2^ii^	0.86	2.57	3.366 (3)	154

Symmetry codes: (i) −*x*+1/2, −*y*+1, *z*; (ii) −*x*+1, *y*, −*z*+1/2.
